# Co-occurrences of substance use and other potentially addictive behaviors: Epidemiological results from the Psychological and Genetic Factors of the Addictive Behaviors (PGA) Study

**DOI:** 10.1556/2006.2020.00033

**Published:** 2020-06-26

**Authors:** Eszter Kotyuk, Anna Magi, Andrea Eisinger, Orsolya Király, Andrea Vereczkei, Csaba Barta, Mark D. Griffiths, Anna Székely, Gyöngyi Kökönyei, Judit Farkas, Bernadette Kun, Rajendra D. Badgaiyan, Róbert Urbán, Kenneth Blum, Zsolt Demetrovics

**Affiliations:** 1 Institute of Psychology, ELTE Eötvös Loránd University, Budapest, Hungary; 2 Doctoral School of Psychology, ELTE Eötvös Loránd University, Budapest, Hungary; 3 Department of Medical Chemistry, Molecular Biology and Pathobiochemistry, Semmelweis University, Budapest, Hungary; 4 International Gaming Research Unit, Psychology Department, Nottingham Trent University, Nottingham, UK; 5 SE-NAP2 Genetic Brain Imaging Migraine Research Group, Hungarian Academy of Sciences, Semmelweis University, Budapest, Hungary; 6 Department of Pharmacodynamics, Faculty of Pharmacy, Semmelweis University, Budapest, Hungary; 7 Nyírő Gyula National Institute of Psychiatry and Addictions, Budapest, Hungary; 8 Department of Psychiatry, Ichan School of Medicine at Mount Sinai, New York, NY, USA; 9 Graduate School of Biomedical Sciences, Western University of Health Sciences, Pomona, CA, USA

**Keywords:** substance use, alcohol use, smoking, cannabis use, behavioral addictions, epidemiology, co-occurrences, gambling, problematic gaming, eating disorder, exercise addiction, trichotillomania, problematic social networking

## Abstract

**Background and aims:**

Changes in the nomenclature of addictions suggest a significant shift in the conceptualization of addictions, where non-substance related behaviors can also be classified as addictions. A large amount of data provides empirical evidence that there are overlaps of different types of addictive behaviors in etiology, phenomenology, and in the underlying psychological and biological mechanisms. Our aim was to investigate the co-occurrences of a wide range of substance use and behavioral addictions.

**Methods:**

The present epidemiological analysis was carried out as part of the Psychological and Genetic Factors of the Addictive Behaviors (PGA) Study, where data were collected from 3,003 adolescents and young adults (42.6% males; mean age 21 years). Addictions to psychoactive substances and behaviors were rigorously assessed.

**Results:**

Data is provided on lifetime occurrences of the assessed substance uses, their co-occurrences, the prevalence estimates of specific behavioral addictions, and co-occurrences of different substance use and potentially addictive behaviors. Associations were found between (i) smoking and problematic Internet use, exercising, eating disorders, and gambling (ii) alcohol consumption and problematic Internet use, problematic online gaming, gambling, and eating disorders, and (iii) cannabis use and problematic online gaming and gambling.

**Conclusions:**

The results suggest a large overlap between the occurrence of these addictions and behaviors and underlies the importance of investigating the possible common psychological, genetic and neural pathways. These data further support concepts such as the Reward Deficiency Syndrome and the component model of addictions that propose a common phenomenological and etiological background of different addictive and related behaviors.

## Introduction

The conceptualization of addictions has changed considerably in the past few years. However, the issue of what to include under the umbrella of addiction is still the focus of both theoretical and empirical research. The fifth edition of the *Diagnostic and Statistical Manual for Mental Disorders* (DSM-5; American Psychiatric Association [APA], 2013) and the eleventh revision of the *International Classification of Diseases* (ICD-11; World Health Organization [WHO], 2018) have addressed the nosological issue of whether ‘addiction’ should include only substance use, or other non-substance related behaviors, as well.

At present, Gambling Disorder is included in the ‘Substance-Related and Addictive Disorders’ category in the DSM-5, and Gambling Disorder and Gaming Disorder are both included in the ICD-11 (American Psychiatric Association [APA], 2013; ICD-11; World Health Organization [WHO], 2018; King et al., 2018; Kiraly & Demetrovics, 2017; Rumpf et al., 2018). Internet gaming disorder [IGD] was included in Section III of the DSM-5 as a potentially addictive behavior to be considered for further research (Griffiths, King, & Demetrovics, 2014a; Király, Griffiths, & Demetrovics, 2015). These fundamental changes in the approach of conceptualizing addictions emphasize that addictions are not always substance-related, and that the characteristics of the behaviors are much more general, therefore applicable to substance and non-substance related disorders (Demetrovics & Griffiths, 2012; Grant, Potenza, Weinstein, & Gorelick, 2010).

Additionally, there are some other behavioral disorders in the DSM-5 and ICD-11 which are categorized in other classes, although they are also occasionally referred to as addictions or could be conceptualized as such (Demetrovics & Griffiths, 2012; Grant et al., 2014, 2010). These behaviors include hoarding disorder, body-focused repetitive behavior disorders (e.g., trichotillomania, excoriation disorder), obsessive-compulsive disorder (in the ‘Obsessive-Compulsive and Related Disorders’ class), and ‘Impulse Control Disorders’, such as pyromania, kleptomania, and compulsive sexual behavior disorder (e.g., Blum, Badgaiyan, & Gold, 2015; Fontenelle, Oostermeijer, Harrison, Pantelis, & Yücel, 2011; Grant; Odlaug, & Potenza, 2007; Kraus, Voon, & Potenza, 2016). Furthermore, there are approaches that focus on the potentially addictive characteristics of eating disorders such as anorexia nervosa, bulimia nervosa, binge eating disorder or becoming obese (e.g., Blum, Thanos, & Gold, 2014b; Cassin & von Ranson, 2007; Davis & Carter, 2009; Davis & Claridge, 1998). There are also other problematic behaviors that are not classified as disorders in the DSM or the ICD, but are often considered as potential behavioral addictions including buying-shopping disorder (i.e., compulsive shopping) (e.g., Müller et al., 2019), exercise addiction (e.g., Archer, Badgaiyan, & Blum, 2017; Berczik et al., 2012; Freimuth, Moniz, & Kim, 2011), social networking addiction (e.g., Andreassen, 2015; Griffiths, Kuss, & Demetrovics, 2014b), and work addiction (e.g., Griffiths, 2011; Sussman, 2012).

There are numerous studies in the literature providing evidence on the relatedness of these potentially addictive behaviors. Epidemiological studies have shown high comorbidities between psychoactive substance use disorders and other potentially addictive behaviors (Di Nicola et al., 2015; Grant, Mancebo, Pinto, Eisen, & Rasmussen, 2006b; Grant & Potenza, 2005; Griffiths & Sutherland, 1998; Griffiths, Wardle, Orford, Sproston, & Erens, 2010; Sussman, Lisha, & Griffiths, 2011; Van Rooij et al., 2014). Similarly, there appears to be overlaps in the underlying psychological mechanisms of these behaviors. It seems that specific personality traits (Andreassen et al., 2013), impulsivity (Walther, Morgenstern, & Hanewinkel, 2012), and motivational factors (Ream, Elliott, & Dunlap, 2011) play an important contributory role in both substance use and other potential behavioral addictions. Furthermore, research from biochemical, neuroimaging, genetic, and treatment perspectives has also suggested a strong neurobiological association between substance use disorders and behavioral addictions (e.g., Blum, Febo, et al., 2014a; Blum et al. 2017; Grant, Brewer, & Potenza, 2006a; Leeman & Potenza, 2013).

The phenomenological description and symptoms of substance use and potentially addictive behaviors appear to share common ground, which is also reflected in the diagnostic criteria of such disorders in both the DSM-5 and ICD-11. In fact, the criteria of substance use disorders were the starting point for developing the criteria for behavioral addictions, such as Gambling Disorder and Gaming Disorder (Petry et al., 2014). There are also a few theoretical models which emphasize the phenomenological and symptomological similarities of different addictions. The Obsessive-Compulsive Spectrum Disorder (OCSD) model (Hollander, 1993; Hollander & Wong, 1995) suggests that disorders from several diagnostic categories share some obsessive-compulsive features. The model is based on a compulsive-impulsive spectrum and proposes that the similarities in phenomenology, etiology, pathophysiology, patient characteristics, and treatment response of clearly distinct disorders are due to these shared obsessive-compulsive aspects. For example, disorders of impulse control are characterized by impulsivity, and lack of control (disinhibition). Affected individuals derive pleasure, arousal and gratification from their impulsive behavior (e.g., gambling addiction, compulsive shopping). Moreover, the Reward Deficiency Syndrome (Blum et al., 1996) hypothesis suggests a common psychological and molecular pathway underlying impulsive, compulsive and addictive behaviors. Blum et al. suggest and confirm in several studies that there is a hypodopaminergic trait that leads to the so-called Reward Deficiency Syndrome (e.g., Blum et al., 2000, 2007; Comings & Blum, 2000). They propose, that defects in the dopaminergic system could have a big impact in developing Reward Deficiency Syndrome and that such individuals are at risk for addictive, impulsive and compulsive behaviors to stimulate the reward cascade. From another perspective, Griffiths (2005) argued in the Component Model of Addictions that all addictions share six basic characteristics (i.e., salience, mood modification, tolerance, withdrawal, conflict, and relapse). These models suggest that there might be common psychological and molecular pathways underlying the similarities in the symptomology, etiology and pathophysiology of different addictive disorders.

The present epidemiological study contributes to the understanding of the association between these behaviors by investigating the co-occurrences of several substance use and potential behavioral addictions utilizing data from the Psychological and Genetic Factors of the Addictive Behaviors (PGA) study (Kotyuk et al., 2019). Although, it is not entirely clear – as it has been shown in the aforementioned literature – which specific disorders should be included in the umbrella term of ‘addiction’, the present study focused on behaviors which in phenomenology or symptomology appear to be related to addiction. Consequently, the selection of examined behaviors is both independent and more wide-ranging than the disorders in current classifications, such as those found in the DSM-5 and ICD-11. In addition to substance use (comprising 14 different substances), the present study also examined the comorbidity of seven potentially addictive behaviors (i.e., Internet use, online gaming, social networking site use, exercising, gambling, hair pulling, and eating disorder).

## Methods

The PGA Study (Kotyuk et al., 2019) is a wide-spectrum national study, where data were collected in four waves from different institutions from a total of 3,003 adolescents and young adults (last year high school students [22%], and college/university students) utilizing a convenience sampling approach. In case of the high schools, research assistants visited classes asking the students to participate, while in case of the college and university students, research assistants recruited the students in dormitories face-to-face to participate in the study. The mean age of the total sample was 21 years (SD = 2.8, min. 18 – max 28 years), with 42.6% of the sample being male. A more detailed description of the sample and the procedure is presented in Kotyuk et al. (2019).

Addictions to both psychoactive substances and behaviors were thoroughly assessed. Fifteen substance use (i.e., nicotine, alcohol, marijuana, synthetic marijuana, amphetamine, cocaine, heroin, lysergic acid diethylamide, a psychedelic drug (LSD), magic mushroom, gamma-hydroxybutyrate, hallucinogenic drug (GHB), mephedrone, steroids, alcohol with drugs, sedative, and other drugs) and seven potentially addictive behaviors (i.e., Internet use, online gaming, social networking site use, exercising, gambling, hair pulling, eating disorder) were assessed.

Lifetime use of psychoactive substances was assessed by a question ‘Have you ever tried alcohol, cigarettes, etc.?’. In case of alcohol drinking and smoking habits, a few follow-up questions were also asked (e.g., ‘How many cigarettes do you smoke a day?’ For full description of the follow-up questions assessing substance use severity, see Supplementary material). Regarding behavioral addictions, the selection of potentially addictive behaviors included in the PGA study was based on prevalence in this young adult population. We wanted to target those potentially addictive behaviors, which are the most frequent among this age group. Thus, the following behaviors were assessed: problematic social media use was assessed using the Bergen Social Media Addiction Scale (BSMAS; Andreassen, Torsheim, Brunborg, & Pallesen, 2012; Banyai et al., 2017), problem gambling was assessed using the Diagnostic Statistical Manual-IV-Adapted for Juveniles (DSM-IV-MR-J; Fisher, 2000), eating disorders were assessed using the SCOFF questionnaire (Morgan, Reid, & Lacey, 1999), exercise addiction was assessed using the Exercise Addiction Inventory (EAI; Griffiths, Szabo, & Terry, 2005; Terry, Szabo, & Griffiths, 2004), hair pulling was assessed using the Massachusetts General Hospital Hairpulling Scale (MGH-HPS; Keuthen et al., 1995), problematic Internet use was assessed using the Problematic Internet Use Questionnaire (PIUQ; Demetrovics, Szeredi, & Rozsa, 2008; Laconi et al., 2019), and problematic gaming was assessed using the Problematic Online Gaming Questionnaire Short-Form (POGQ-SF; Papay et al., 2013). Psychometric properties of the questionnaires were adequate. For a detailed description of the PGA study protocol and the psychometric properties of all the instruments used see Kotyuk et al. (2019).

To establish prevalence estimates for the problematic occurrence of these behaviors, cut-off thresholds were used as originally described in the PGA study protocol (Kotyuk et al., 2019). Problematic behaviors were defined as: 19 points or more (out of 30) for the BSMAS (Banyai et al., 2017); 4 or more (out of 9) for the DSM-IV-MR-J (Fisher, 2000); 2 or more (out of 5) for the SCOFF questionnaire (Luck et al., 2002; Morgan et al., 1999); 15 or more (out of 30) for the PIUQ (Demetrovics et al., 2016); 32 or more (out of 48) for the POGQ-SF (Papay et al., 2013); and 24 or more (out of 30) for the EAI (Griffiths et al., 2015; Mónok et al., 2012). Although it has been suggested that the cut-off for clinical significance on the MGH-HPS measure is 17 or more (out of 28; Keuthen et al., 2007; Woerner, Selles, De Nadai, Salloum, & Storch, 2017), as far as we can tell, this cut-off threshold is a theoretical suggestion, based on the mean MGH-HPS score (17 ± 5.07) of a sample of Internet surveyed self-reported hairpullers reported by Keuthen et al. (2007). Thus, further studies are needed to validate this cut-off score. In the present analysis cut-off threshold for problematic hair pulling behavior was used as mean score plus two times the standard deviation: 1.43 + 2 × 4.01 = 9.45. In conclusion, participants scoring 10 or higher on the MGH-HPS scale were considered as problematic hairpullers.

## Statistical analysis

Description of substance use rates was carried out by using a binary lifetime use (yes or no) variable. Severity of substance use was described by analyzing more detailed questions regarding the frequency of substance usage or in case of potentially addictive behaviors, by the appropriate psychometric scales. Frequencies of potentially addictive behaviors were assessed by specific questions regarding the amount of time spent on each specific activity. Severity of potentially addictive behaviors was calculated by using the cut-off thresholds of the assessed behavioral addiction psychometric instruments (outlined in the previous section). Co-occurrences of substance use types, as well as co-occurrences of potentially addictive behaviors were tested with chi-square analysis. Phi coefficients were also calculated to test the strength of these relationships. Odds ratios with 95% confidence intervals were also calculated to present risk estimate. For the analysis of the possible association between regular substance use and the severity of potentially addictive behaviors, specific behavioral addiction scale mean scores by substance users and non-users were compared. In these analyses, only the most frequent substances of the present sample (nicotine [cigarettes], alcohol, marijuana) were included, and independent sample *t*-tests were used to test for differences in the potentially addictive behavior scales' mean scores by regular and non-regular psychoactive substance users. The effect sizes are expressed in Cohen's *d* statistics which reflect the explained variance in the dependent variable due to the grouping variable. False positive results were ruled out by Bonferroni correction for multiple testing (Bonferroni, 1936; Miller, 1981).

### Ethics

The study protocol was designed in accordance with guidelines of the Declaration of Helsinki, and was approved by the Scientific and Research Ethics Committee of the Medical Research Council (ETT TUKEB). Recruitment started with contacting the heads of several high schools and universities to acquire institutional consents. Afterwards, participants were recruited on a voluntary basis, and provided written informed consent.

## Results

### Sex and age differences in substance occurrences, and severity of potentially addictive behaviors

First potential differences among males and females were tested in case of the assessed lifetime substance use categories (see results in Supplementary Table 1). Lifetime occurrence of marijuana (*P* < 0.001), synthetic marijuana (*P* < 0.001), amphetamine (*P* < 0.001), cocaine (*P* < 0.001), heroin (*P* = 0.007), LSD (*P* = 0.001), magic mushroom (*P* < 0.001), GHB (*P* < 0.001), mephedrone (*P* < 0.001), steroids (*P* = 0.001), other drugs (*P* < 0.001) and ‘unknown type of drug’ (*P* = 0.017) was significantly higher in case of males as compared to the females. Lifetime occurrence of sedatives was significantly higher in case of females as compared to males (*P* < 0.001). In case of the other substances no significant differences among the two sexes were observed. In the case of the potentially addictive behaviors, males showed a significantly higher mean score on the POGQ (*P* < 0.001), on the EAI (*P* < 0.015) and on the Diagnostic Statistical Manual-IV-Adapted for Juveniles questionnaire measuring gambling (*P* < 0.001), as compared to females. Females showed significantly higher mean scores then males on the SCOFF (*P* < 0.001) and on the BSMAS questionnaire (*P* < 0.001) measuring eating disorders and social networking site use habits. No differences were observed in case of males and females by the PIUQ and MGH-HPS questionnaires. The detailed results can be found in Supplementary Table 2.

As regards to age, out of the assessed lifetime substance use categories, lifetime alcohol (*P* = 0.002), amphetamine (*P* = 0.035), cocaine (*P* = 0.038), LSD (*P* = 0.007), magic mushroom (*P* = 0.025), and GHB (*P* = 0.046) showed a significant association with age. However, the effect sizes (*η*^2^ = 0.003; *η*^2^ = 0.002; *η*^2^ = 0.001; *η*^2^ = 0.002; *η*^2^ = 0.002; *η*^2^ = 0.001, respectively), and the mean age differences amongst the lifetime ever and never users were negligible (e. g. mean age for lifetime amphetamine users: 22.52 ± 3.1 years, mean age for those who never used amphetamine in their lifetime: 22.12 ± 3.1 years). In case of the assessed potentially addictive behaviors, correlational analysis showed a negative relationship between age and SCOFF (*r* = 0.051 *P* = 0.005), PIUQ (*r* = 0.093 *P* < 0.001), and EAI scores (*r* = 0.066 *P* < 0.001). However, the Pearson correlation values were rather small.

### Substance occurrences, co-occurrences and severity

Table 1A and B summarizes lifetime occurrences of the assessed 15 types of psychoactive substances and the co-occurrences of lifetime usage of these substances. As it can be seen from Table 1A and B, most participants had previously drunk alcohol (94.9%) and smoked cigarettes (69.1%) at some point in their life. One-third of the sample had tried marijuana (33.0%). Other lifetime psychoactive substance use was low: synthetic marijuana (8.6%), sedatives (7.7%), alcohol mixed with drugs (6.3%), and amphetamines (5.8%). Lifetime use of all other substances was below 5%.

**Table 1A.** Lifetime occurrence and co-occurrence of psychoactive substance use

	Cigarettes (*N* = 2,982)	Alcohol (*N* = 2,990)	Marijuana (*N* = 2,990)	Synthetic marijuana (*N* = 1723)	Amphetamine (*N* = 2,980)	Cocaine (*N* = 2,983)	Heroin (*N* = 2,979)	LSD (*N* = 2,980)
Cigarettes	69.1%	3.3 [2.4–4.6]	12.6 [9.5–16.7]	20.9 [7.7–56.7]	13.3 [5.9–30.2]	11.7 [2.8–48.1]	9.1 [1.2–67.6]	10.3 [2.5–42.5]
Alcohol	**67.1%** 0.136**	94.9%	19.7 [7.3–53.4]	7.3 [1.0–52.9]	nc1	2.9 [0.4–21.0]	1.1 [0.1–8.1]	2.5 [0.3–18.3]
Marijuana	**31.1%** 0.382**	**33%** 0.150**	33.0%	18.5 [11.2–30.6]	68.8 [30.4–156.0]	114.8 [15.8–831.2]	19.9 [4.6–85.7]	99.2 [13.7–720.3]
Synthetic marijuana	**8.4%** 0.202**	8.6% 0.056*	**7.6%** 0.356**	8.6%	26.5 [15.7–44.8]	20.3 [9.1–45.2]	42.1 [11.6–152.5]	48.1 [19.2–120.3]
Amphetamine	**5.6%** 0.147**	5.8% 0.057*	**5.6%** 0.338**	**2.6%** 0.409**	5.8%	214.1 [83.7–547.9]	33.8 [13.3–85.8]	87.8 [41.5–185.8]
Cocaine	**1.7%** 0.079**	1.8% 0.020	**1.8%** 0.189**	**1%** 0.245**	**1.6%** 0.487**	1.8%	82.3 [33.1–205.1]	52.9 [26.9–104.3]
Heroin	0.7% 0.048*	0.7% 0.001	**0.6%** 0.104**	**0.6%** 0.226**	**0.4%** 0.212**	**0.4%** 0.333**	0.7%	91.8 [36.6–230.2]
LSD	**1.5%** 0.073**	1.5% 0.017	**1.5%** 0.176**	**1.3%** 0.330**	**1.2%** 0.406**	**0.6%** 0.353**	**0.4%** 0.347**	1.6%
Magic mushrooms	1.6% 0.065**	1.7% 0.030	**1.6%** 0.182**	**1.1%** 0.261**	**1.3%** 0.397**	**0.6%** 0.358**	**0.2%** 0.221**	**0.8%** 0.497**
GHB	1.4% 0.070**	1.5% 0.016	**1.4%** 0.166**	**0.6%** 0.217**	**1.1%** 0.379**	**0.5%** 0.320**	**0.3%** 0.259**	**0.6%** 0.386**
Mephedrone	**2.9%** 0.109**	3.1% 0.041*	**2.9%** 0.229**	**1.0%** 0.279**	**2.4%** 0.553**	**0.8%** 0.351**	**0.3%** 0.175**	**0.8%** 0.358**
Steroids	**2.3%** 0.072**	2.4% −0.001	**1.7%** 0.109**	**0.8%** 0.157**	**0.8%** 0.175**	**0.4%** 0.192**	**0.3%** 0.191**	**0.4%** 0.204**
Alcohol with drugs	**5.8%** 0.126**	6.3% 0.048*	**4.7%** 0.227**	**1.9%** 0.214**	**1.7%** 0.250**	**0.7%** 0.188**	**0.7%** 0.195**	**0.9%** 0.257**
Sedatives	**6.5%** 0.090**	7.6% 0.033	**4.3%** 0.141**	**1.9%** 0.176**	**1.5%** 0.178**	**0.7%** 0.154**	**0.4%** 0.142**	**0.6%** 0.145**
Other drugs	1.9% 0.056*	2.1% 0.004	**1.8%** 0.157**	**1.0%** 0.196**	**0.9%** 0.246**	**0.4%** 0.201**	**0.3%** 0.257**	**0.7%** 0.365**
unknown type of drug^a^	**1.7%** 0.073**	1.8% 0.019	**1.3%** 0.111**	0.4% 0.081*	**0.7%** 0.203**	**0.3%** 0.163**	**0.3%** 0.245**	**0.5%** 0.275**

*Notes.* Numbers of valid answers by each substance use are presented in the table header. Lifetime use of each substance is presented in the diagonal (% based on valid answers). Co-occurrences of specific substance uses are presented in the cells below the diagonal (% of overlap calculated by number of cases with both characteristics divided through all cases, based on the valid answers of the total sample) with the corresponding Phi coefficient. * Phi correlation is significant at the 0.05 level (2-tailed). ** Phi correlation is significant at the 0.001 level (2-tailed). Chi-square tests where the level of significance is smaller than the Bonferroni correction significance level for 130 analysis (*P* < 0.000394) are marked in bold. Odds Ratios and 95% confidence intervals are presented above the diagonal. Most missing data occurred by the ‘synthetic marijuana’ question (49.4%). In all other cases the rate of missing data was less than 15%.

nc1: non-calculable due to the zero frequency in one cell, only estimated with replacing missing cell with 1: OR = 9.8 [1.4–70.6].

nc2: non-calculable due to the zero frequency in one cell, only estimated with replacing missing cell with 1: OR = 2.7 [0.4–20.0].

nc3: non-calculable due to the zero frequency in one cell, only estimated with replacing missing cell with 1: OR = 5.1 [0.7–36.6].

^a^The ‘unknown type of drug’ category represent participants' answers where they do not know what type of substance they used.

**Table 1B.** Lifetime occurrence and co-occurrence of psychoactive substance use

	Magic mushrooms (*N* = 2,981)	GHB (*N* = 2,981)	Mephedrone (*N* = 2,982)	Steroids (*N* = 2,973)	Alcohol with drugs (*N* = 2,978)	Sedatives (*N* = 2,763)	Other drugs (*N* = 2,891)	unknown type of drug (*N* = 2,572)
Cigarettes	5.3 [1.9–14.6]	9.6 [2.3–39.7]	20.3 [5.0–82.7]	3.9 [1.9–8.2]	5.2 [3.1–8.7]	2.4 [1.7–3.4]	2.8 [1.4–5.8]	6.7 [2.1–21.7]
Alcohol	nc2	2.4 [0.3–17.5]	nc3	1.0 [0.4–2.7]	5.3 [1.3–21.4]	2.1 [0.9–4.8]	1.1 [0.4–3.6]	2.5 [0.3–18.4]
Marijuana	105.9 [14.6–768.0]	46.1 [11.2–190.9]	31.7 [13.8–72.9]	3.9 [2.4–6.2]	6.6 [4.7–9.2]	2.8 [2.1–3.7]	9.5 [5.1–18.0]	5.3 [2.8–10.1]
Synthetic marijuana	21.1 [9.8–45.3]	31.5 [9.9–100.3]	40.7 [14.8–112.1]	7.9 [3.8–16.2]	6.5 [4.1–10.3]	4.7 [3.0–7.3]	10.2 [5.1–20.3]	4.1 [1.6–10.7]
Amphetamine	67.6 [34.5–132.3]	70.4 [34.1–145.6]	99.9 [58.5–170.7]	8.4 [5.0–14.2]	9.0 [6.2–13.0]	5.3 [3.7–7.8]	15.7 [9.2–26.8]	13.1 [7.0–24.5]
Cocaine	52.2 [26.9–101.4]	44.4 [22.2–88.9]	40.8 [22.5–74.1]	15.5 [7.9–30.6]	11.3 [6.4–20.2]	8.1 [4.6–14.4]	19.4 [9.2–40.7]	14.6 [6.4–33.4]
Heroin	39.3 [14.9–103.7]	51.4 [20.0–131.8]	22.1 [8.9–54.9]	26.5 [10.6–66.0]	26.4 [10.8–64.7]	13.9 [5.9–33.2]	51.2 [19.6–134.0]	46.4 [16.8–128.2]
LSD	126.4 [62.9–254.3]	69.4 [34.3–140.2]	46.0 [24.7–85.8]	17.9 [9.0–35.6]	21.4 [11.8–38.9]	8.0 [4.4–14.6]	56.4 [28.4–112.1]	33.9 [15.8–72.8]
Magic mushrooms	1.7%	62.5 [31.2–125.0]	41.7 [22.6–76.8]	11.4 [5.5–23.8]	12.6 [7.0–22.6]	7.5 [4.1–13.6]	35.7 [17.3–73.8]	20.2 [9.0–45.6]
GHB	**0.6%** 0.373**	1.5%	61.4 [32.3–116.6]	24.3 [12.4–47.7]	9.0 [4.8–16.8]	7.8 [4.2–14.5]	32.7 [16.0–66.9]	21.7 [9.6–49.2]
Mephedrone	**0.8%** 0.347**	**0.9%** 0.396**	3.1%	13.7 [7.7–24.2]	8.1 [5.1–12.9]	4.8 [3.0–7.9]	22.9 [12.7–41.4]	15.0 [7.4–30.5]
Steroids	**0.3%** 0.148**	**0.5%** 0.245**	**0.6%** 0.211**	2.6%	7.5 [4.5–12.5]	4.1 [2.3–7.0]	18.6 [10.0–34.5]	18.6 [9.2–37.4]
Alcohol with drugs	**0.7%** 0.196**	**0.5%** 0.150**	**1.0%** 0.187**	**0.8%** 0.163**	6.3%	11.3 [8.1–15.8]	9.1 [5.3–15.7]	15.1 [8.2–27.7]
Sedatives	**0.6%** 0.141**	**0.6%** 0.140**	**0.8%** 0.127**	**0.6%** 0.099**	**2.5%** 0.312**	7.7%	3.8 [2.0–6.9]	7.3 [3.9–13.7]
Other drugs	**0.5%** 0.280**	**0.5%** 0.272**	**0.7%** 0.271**	**0.6%** 0.235**	**0.7%** 0.176**	**0.5%** 0.085**	2.2%	26.2 [12.1–56.4]
unknown type of drug^a^	**0.4%** 0.199**	**0.3%** 0.205**	**0.5%** 0.194**	**0.5%** 0.220**	**0.8%** 0.226**	**0.6%** 0.143**	**0.4%** 0.241**	1.8%

*Notes.* Numbers of valid answers by each substance use are presented in the table header. Lifetime use of each substance is presented in the diagonal (% based on valid answers). Co-occurrences of specific substance uses are presented in the cells below the diagonal (% of overlap calculated by number of cases with both characteristics divided through all cases, based on the valid answers of the total sample) with the corresponding Phi coefficient. * Phi correlation is significant at the 0.05 level (2-tailed). ** Phi correlation is significant at the 0.001 level (2-tailed). Chi-square tests where the level of significance is smaller than the Bonferroni correction significance level for 130 analysis (*P* < 0.000394) are marked in bold. Odds Ratios and 95% confidence intervals are presented above the diagonal. Most missing data occurred by the ‘synthetic marijuana’ question (49.4%). In all other cases the rate of missing data was less than 15%.

nc1: non-calculable due to the zero frequency in one cell, only estimated with replacing missing cell with 1: OR = 9.8 [1.4–70.6].

nc2: non-calculable due to the zero frequency in one cell, only estimated with replacing missing cell with 1: OR = 2.7 [0.4–20.0].

nc3: non-calculable due to the zero frequency in one cell, only estimated with replacing missing cell with 1: OR = 5.1 [0.7–36.6].

^a^The ‘unknown type of drug’ category represent participants' answers where they do not know what type of substance they used.

Lifetime co-occurrences of the examined substances were also calculated, and percentages of lifetime co-occurrence based on the total sample (% of co-occurrences were calculated by number of cases with both characteristics divided through all cases) are presented below the diagonal in Table 1A and B. As expected, co-occurrence was the highest between alcohol and cigarette smoking (67.1%), moderate for marijuana consumption and cigarette smoking (31.1%), and moderate for marijuana and alcohol consumption (33.0%). In all other cases, the co-occurrence was below 10%. Table 1A and B also summarizes the level of significance of the Chi-square tests, marked by bold where level of significance was smaller than the Bonferroni correction level for 130 analysis (*P* < 0.000394). Phi coefficients were also calculated to assess the association between two binary variables. The highest Phi coefficient was observed in the co-occurrence analysis of mephedrone and amphetamine (Φ = 0.55; *P* < 0.000394), meaning that those who have tried mephedrone were likely to have tried amphetamine too. The second highest Phi coefficient was observed in case of magic mushrooms and LSD (Φ = 0.50; *P* < 0.000394), followed by cocaine and amphetamine (Φ = 0.49; *P* < 0.000394), amphetamine and synthetic marijuana (Φ = 0.41; *P* < 0.000394), and GHB and LSD (Φ = 0.39; *P* < 0.000394). These Phi coefficients suggest a moderate relationship in case of these co-occurring illicit drugs, while the relationships between the use of other substances were either weak or negligible (average Phi coefficient was 0.201 ± 0.116). Odds ratio were the lowest in case of alcohol consumption and the ‘other drugs’ category (OR = 1.1; 95% CI 0.4–3.6), and highest in case of trying out amphetamine and cocaine (OR = 214.1; 95% CI 83.7–547.9). There seems to be a pattern in odds ratios in Table 1: ORs for licit drugs (alcohol and cigarettes) seem to be lower, while in case of the illicit drugs ORs are higher, suggesting that lifetime consumption of one type of illicit substance also increases the likelihood of trying other illicit substances.

As expected, the most commonly used psychoactive substance in the sample was alcohol, nicotine (cigarettes), and cannabis consumption (see Table 1A and B). When assessing cigarette smoking habits in more details, it was found that 59.7% of the sample did not smoke at the time of the data collection, 23.1% smoked occasionally, and 17.1% smoked regularly. Missing data occurred in 0.1% of the sample (*n* = 4). A small minority of the sample (7%) reported smoking more than 10 cigarettes a day. Two-thirds of participants tried smoking cigarettes for the first time in their lives after the age of 10 years (65%).

For alcohol consumption, 98.2% of the sample (*n* = 2,948) gave valid answer for the question of “How often did you consume alcohol in the past 30 days?”, of which 14.0% did not drink during the month preceding the data collection, 58.0% drank one to three times, 21.7% drank 4–9 times, 5.1% drank 10–19 times, 0.9% reported drinking not every day, but more than 20 days a month, and 0.2% (seven participants) reported drinking every day. When screening for drinking more than six units of alcohol in the past 30 days, 97.8% of the sample gave valid answers (*n* = 2,937), of which 60.2% reported that they did not drink six units of alcohol in the month preceding the data collection, 32.3% reported that it happened one to three times, 6.5% reported 4–9 times, 0.9% reported 10–19 times, 0.1% reported not every day, but more than 20 days in the month, and one participant reported drinking at least six units of alcohol every day in the past 30 days. Over four-fifths of the sample reported their first consumption of alcohol occurring after the age of 10 years (83%).

For marijuana use frequency, participants were asked how often they used marijuana in the past 30 days. However, participants were only asked to answer this question if they had used marijuana in their lifetime. Therefore, valid answers were obtained from 33.0% of the sample (*n* = 992), of which 70.0% reported no use of marijuana in 30 days preceding the data collection, 22.0% reported consumption one to three times, 4.3% reported 4–9 times, 2.3% reported 10–19 times, 0.8% reported not every day, but more than 20 days in a month, and 0.6% reported daily consumption in the 30 days preceding the data collection. Mean Cannabis Abuse Screening Test score in the present sample was 6.9 ± 2.2 (out of 30) with the scale scores between 6 and 30 meaning that it was fairly low in the present sample. Furthermore, all participants who have tried marijuana reported their first use after the age of 10 years.

### Occurrences, co-occurrences, and severity of potentially addictive behaviors

The present study assessed frequency and time spent with different potentially addictive behaviors (Internet use, gaming, social networking, gambling, exercising, and hair pulling). The use of social networking sites and Internet in general was relatively prevalent in the present sample. Around 10% spent more than eight hours a day on social networking sites or on the Internet. Gaming for long hours a day was quite rare, and most of the sample (65.9%) had never played online videogames. However, the question concerning videogame play only inquired about online gaming, and there were no data collected on offline gaming. Regarding gambling, 2,978 participants (99.2%) had valid data from which 2,145 reported that they had gambled in their lives (72.0%). The rest (28.0%, *n* = 833) had never gambled. Most participants (*N* = 2,989) gave valid answers for the frequency of intense exercise question (99.5%), of which 4.5% reported exercising intensely every day, 12.4% reported four to six days a week, 27.3% reported two to three times a week, 20.9% reported once every week, 10.9% monthly, 15.6% occasionally, and 8.4% never. Trichotillomania was also assessed, but only included in the third and fourth data collection waves, therefore valid answers were received from 1730 participants for the prevalence of hair pulling. Of these, 70.1% reported that they had never pulled their hair knowingly, 9.9% reported that it happened to them, but more than a year ago, 6.1% reported they pulled their hair in the past year, but not in the past month, 4.3% reported hair pulling in the past month, but not in the past seven days, and 9.7% reported hair pulling in the past seven days.

As noted above, the severity of these potentially addictive behaviors was assessed by psychometrically sound screening instruments. To assess the problematic occurrence of these behaviors the number of participants above each scale's cut-off threshold were calculated. The diagonal of Table 2 summarizes the occurrences of potentially addictive behaviors defined by the cut-off thresholds of the scales. For problematic Internet use, the mean PIUQ score was 10.2 ± 3.6 (out of 30). A total of 13.3% were classed as at-risk problematic Internet users (scoring more than 15). For problematic gaming, the mean POGQ-SF score was 15.8 ± 6.3 (out of 48). A total of 4.0% were classed as at-risk problematic gamers. For social media addiction, the mean BSMAS score in the present sample was 9.6 ± 3.8 (out of 30). A total 3.2% were considered as being at risk of social media addiction (scoring 19 or more). For exercise addiction, the mean EAI score was 12.4 ± 5.0 (out of 30). A total of 2.3% were classed as being at risk of exercise addiction (scoring 24 or more). For problem gambling, the mean DSM-IV-MR-J score was 0.31 ± 0.8 (out of 9). A total of 1.7% were classed as problem gamblers (scoring 4 or more). For hair pulling, the mean MGH-HPS score was 1.4 ± 4.0 (out of 28). A total of 4% were classed as problematic hair pullers (scoring two standard deviations above the mean score). For eating disorders, the mean SCOFF score was 0.7 ± 1.0 (out of 5). A total of 19.6% were classed as having an eating disorder (scoring 2 or more).

**Table 2.** Occurrence and co-occurrence of potential behavioral addictions defined by the cut-off thresholds of the appropriate scales

	Problematic Internet use	Problematic online gaming	Problematic use of social networking sites^a^	Exercise addiction	Problematic gambling	Trichotillomania	Eating disorder
Problematic Internet use	13.3% (*n* = 396)	13.7 [9.2–20.5]	35.2 [17.0–73.0]	1.4 [0.7–2.6]	3.5 [1.9–6.3]	2.5 [1.7–3.9]	2.3 [1.8–2.9]
Problematic online gaming	**2.5%** **0.302∗∗∗**	4.0% (*n* = 113)	3.4 [1.4–8.4]	2.9 [1.3–6.5]	12.2 [6.4–23.2]	3.1 [1.7–5.9]	1.5 [1.0–2.3]
Problematic use of social networking sites^a^	**2.7%** **0.351∗∗**	0.4% 0.072∗	3.2% (*n* = 55)	4.8 [1.6–14.2]	6.0 [1.7–21.2]	3.7 [1.9–7.4]	4.0 [2.3–6.8]
Exercise addiction	0.4% 0.017	0.2% 0.051∗	**0.2%** **0.075∗**	2.3% (*n* = 70)	8.9 [4.0–19.7]	1.1 [0.3–3.5]	2.1 [1.2–3.4]
Problematic gambling	**0.6%** **0.081∗∗**	**0.5%** **0.182∗∗**	**0.4%** **0.076∗**	**0.2%** **0.119∗∗**	1.7% (*n* = 50)	3.4 [1.4–8.2]	1.2 [0.6–2.3]
Trichotillomania	**1.1%** **0.082∗∗**	**0.4%** **0.070∗∗**	**0.6%** **0.095∗∗**	0.1% 0.002	0.4% 0.054∗	4.0% (*n* = 120)	1.9 [1.2–2.8]
Eating disorder	**4.4%** **0.133∗∗**	1.1% 0.036	**1.5%** **0.128∗∗**	0.8% 0.052∗	1.1% 0.009	**1.2%** **0.056∗**	19.6% (*n* = 581)

*Notes.* Occurrences of potentially addictive behaviors defined by the cut-off thresholds of the appropriate scales are presented on the diagonal. Co-occurrences of these behaviors are presented in cells below the diagonal (% of overlap calculated by number of cases with both characteristics divided through all cases, based on valid answers), with the corresponding Phi coefficient values. ∗ Phi correlation is significant at the 0.05 level (2-tailed). ∗∗ Phi correlation is significant at the 0.001 level (2-tailed). Chi-square tests where the level of significance is smaller than the Bonferroni correction level for 21 analysis (*P* < 0.00243) are marked in bold. Odds Ratios and 95% confidence intervals are presented above the diagonal.

a Social networking use was only assessed from the 3rd data collection wave.

With regards to the co-occurrences (cells below the diagonal in Table 2), many significant co-occurrences were observed (based on the number of analysis [21], the corrected level of significance was 0.00243). For example, problematic Internet users were more likely to have an online gaming problem, and problematic social media use. These co-occurrences were present not only in online activities, but also in other potentially addictive behaviors. These results suggest an overlap between occurrences of different types of problematic behaviors. However, based on the Phi coefficient values, the degree of these relationships was relatively small, and in many cases negligible (Φ range between 0.002–0.351; average Φ was 0.092 ± 0.088). With regards to odds ratios, the lowest OR was observed in case of problematic gambling and eating disorders (OR = 1.2; 95% CI 0.6–2.3), and the highest OR was between problematic Internet use and problematic use of social networking sites (OR = 35.2; 95% CI 17.0–73.0). Risk calculations for these problematic behaviors showed that the odds of having problems amongst different types of online activities was in most cases higher when compared to the further tested behaviors (e.g., eating disorder).

### Associations between substance use and severity of potentially addictive behaviors

The next analysis compared the mean scale scores of potentially addictive behaviors among regular and non-regular substance users. For this analysis only the three most common types of substance use were examined (i.e., nicotine [cigarette], alcohol, and marijuana consumption). Results of the independent sample *t*-tests are presented in Table 3A and B. Based on the number of analysis (21), the corrected level of significance was 0.00243. There was a significant association between smoking and the severity of problematic Internet use [*t* (2,282) = 3.064, *P* = 0.0022, Cohen's *d* = 0.168]. Smokers had a lower PIUQ mean score compared to non-smokers. There was also a significant association between smoking and exercise addiction [*t* (2,275) = 4.351, *P* < 0.00243, Cohen's *d* = 0.218]. Regular smokers reported lower mean score on the EAI compared to non-smokers. The association between smoking and gambling was also significant [*t* (2,269) = −2.440, *P* = 0.0148, Cohen's *d* = 0.133], in this case regular smoker participants showed a higher mean score on the DSM-IV-MR-J as compared to non-smokers. However, this association did not survive the correction for multiple testing. Additionally, there was a significant association between smoking and the severity of eating disorders [*t* (2,283) = −3.617, *P* < 0.00243, Cohen's *d* = 0.176]. Regular smokers reported a higher mean score on the SCOFF compared to non-smokers.

**Table 3A.** Severity of potential behavior addictions by regular psychoactive substance users and non-users

	Problematic Internet Use Questionnaire (PIUQ)			Problematic Online Gaming Questionnaire Short-Form (POGQ-SF)			Bergen Social Media Addiction Scale (BSMAS)^a^		
	mean ± SD	*P*	Cohen's d	mean ± SD	*P*	Cohen's d	mean ± SD	*P*	Cohen's d
*Nicotine*									
Self report regular smoker (*n* = 513)	**9.7 ± 3.5**	0.002*	**0.168**	15.7 ± 6.2	0.420	0.042	9.5 ± 3.6	0.617	0.039
Self report non-smoker (*n* = 1794)	**10.2 ± 3.6**			16.0 ± 6.4			9.4 ± 3.7		
*Alcohol*									
Drunk 3 or more times in the past 30 days (*n* = 284)	**11.2 ± 4.0**	<0.001*	**0.286**	**17.1 ± 8.0**	<0.001*	**0.207**	**10.6 ± 4.3**	<0.001*	**0.261**
Drunk less than 3 times in the past 30 days (*n* = 2,551)	**10.2 ± 3.6**			**15.7 ± 6.0**			**9.5 ± 3.7**		
*Marijuana*									
Past month marijuana users (*n* = 257)	10.6 ± 3.6	0.096	0.110	16.6 ± 7.0	0.031*	0.136	9.8 ± 3.5	0.391	0.076
Non-users (*n* = 2,733)	10.2 ± 3.6			15.7 ± 6.2			9.6 ± 3.8		

*Notes.* * independent sample *t*-test is significant at the 0.05 level. *t*-tests where the level of significance is smaller than the Bonferroni correction level for 21 analysis (*P* < 0.00243) are marked in bold.

^a^Social networking use was only assessed from the third data collection wave. ± = standard deviation.

**Table 3B.** Severity of potential behavior addictions by regular psychoactive substance users and non-users

	Exercise Addiction Inventory (EAI)			Diagnostic Statistical Manual-IV-Adapted for Juveniles (DSM-IV-MR-J)			The Massachusetts General Hospital Hairpulling Scale (MGH-HPS)			SCOFF Questionnaire eating disorder questionnaire		
	mean ± SD	*P*	Cohen's d	mean ± SD	*P*	Cohen's d	mean ± SD	*P*	Cohen's d	mean ± SD	*P*	Cohen's d
*Nicotine*												
Self report regular smoker (*n* = 513)	**11.4 ± 4.8**	<0.001*	**0.218**	0.3 ± 0.8	0.015*	0.123	1.3 ± 3.7	0.571	0.047	**0.8 ± 1.0**	<0.001*	**0.176**
Self report non-smoker (*n* = 1794)	**12.5 ± 5.0**			0.2 ± 0.9			1.4 ± 4.0			**0.6 ± 0.9**		
*Alcohol*												
Drunk 3 or more times in the past 30 days (*n* = 284)	12.5 ± 5.1	0.753	0.020	**0.5 ± 1.1**	<0.001*	**0.299**	1.9 ± 4.7	0.083	0.133	**1.0 ± 1.1**	<0.001*	**0.320**
Drunk less than 3 times in the past 30 days (*n* = 2,551)	12.4 ± 5.0			**0.2 ± 0.8**			1.4 ± 3.9			**0.7 ± 0.9**		
*Marijuana*												
Past month marijuana users (*n* = 257)	12.2 ± 5.1	0.511	0.047	**0.5 ± 1.1**	<0.001*	**0.267**	1.3 ± 4.2	0.759	0.027	0.7 ± 1.0	0.505	0.043
Non-users (*n* = 2,733)	12.5 ± 5.0			**0.2 ± 0.8**			1.4 ± 4.0			0.7 ± 1.0		

*Notes.* * independent sample *t*-test is significant at the 0.05 level. *t*-tests where the level of significance is smaller than the Bonferroni correction level for 21 analysis (*P* < 0.00243) are marked in bold.

With regards to alcohol consumption, there were significant associations with the severity of problematic Internet use [*t* (2,799) = −4.746, *P* < 0.00243, Cohen's *d* = 0.286], problematic gaming [*t* (2,693) = −3.706, *P* < 0.00243, Cohen's *d* = 0.207], social media addiction [*t* (1,671) = −3.406, *P* < 0.00243, Cohen's *d* = 0.261], problem gambling [*t* (2,795) = −4.744, *P* < 0.00243, Cohen's *d* = 0.299], and eating disorders [*t* (2,803) = −5.514, *P* < 0.00243, Cohen's d = 0.320]. In all cases, regular alcohol users (defined by drinking three or more times in the past 30 days) showed higher mean scores on the screening instruments compared to non-regular alcohol users.

With regard to marijuana consumption, there was a significant association with the severity of gambling [*t* (2,943) = −4.047, *P* < 0.00243, Cohen's *d* = 0.267], where past month marijuana users showed a higher mean score on the DSM-IV-MR-J scale as compared to non-users. Further nominally significant associations did not survive the correction for multiple testing based on Bonferroni corrections. Overall, these results suggest a large overlap between the different types of substance usage and potentially addictive behaviors. However, the effect sizes of group differences were typically small or negligible (average Cohen's *d* was 0.148 ± 0.105; see detailed Cohen's *d* values in Table 3A and B).

## Discussion

The present study provided occurrence rates of various addictive behaviors among the Hungarian PGA sample comprising 3,003 young adults. Lifetime usage and co-occurrences were provided for 15 types of psychoactive substance use. Characteristics of lifetime use of substances in the present study were similar to those expected among this age population. Alcohol consumption, cigarette smoking, and marijuana consumption were the most commonly tried substances. Lifetime use of the other 11 psychoactive substances were low (below 10%), but not negligible.

These occurrence rates are similar as previously reported in the literature. For example, a study examining alcohol consumption habits and drinking motives in 13 European countries found that the percentage of students who have been drunk at least once in their lifetime is 81.3% in Hungary based on data of 17 to 19-year-olds (Kuntsche et al., 2014). Furthermore, the European School Survey Project on Alcohol and Other Drugs (ESPAD) survey (Hibell et al., 2009) reported that 55–64% of the asked Hungarian students consumed alcohol during the last 30 days, 25–34% reported cigarette use during the last 30 day; 3–5% reported use of marijuana or hashish in the last 30 days; and 6–15% reported lifetime use of any illicit drug, 9–10% reported lifetime use of tranquillizers or sedatives without a prescription. These reports show similar patterns: alcohol consumption is the most frequent in the Hungarian adolescent population, followed by nicotine, marijuana, and illicit drugs. However, since the PGA study is a convenience sample, interpretation of the presented occurrence rates should be handled with caution.

An interesting pattern also emerged when analyzing the co-occurrence rates of lifetime psychoactive substance use. The analysis presented in Table 1A and B may indicate two clusters in the co-occurrence estimates: licit drugs (alcohol, nicotine, and – although it is not licit in Hungary – marijuana) seems to co-occur frequently, creating one cluster, and there seems to be a separate cluster, comprising illicit drugs: in this case, the co-occurrence rates are smaller, but the odds ratios are higher, suggesting that lifetime usage of one of the illicit substances associates with higher odds of lifetime usage of another illicit substance. Furthermore, it seems that lifetime usage of licit substances, especially alcohol, but in many cases cigarette as well does not show a significant difference amongst the lifetime users and not users of illicit substances (Table 1). For example, in case of the co-occurrence analysis of lifetime alcohol and lifetime cocaine usage, the frequency of lifetime cocaine users were similar in the lifetime alcohol consumers and in those who never tried alcohol in their life. These results suggest, that lifetime illicit substance usage is rather independent from the lifetime usage of the assessed licit substances (alcohol and cigarettes). A German study analyzing the patterns of licit and illicit substance use amongst university students found similar results (Schilling et al., 2017): they identified six clusters, where they found combinations of licit substance user groups, and illicit drug users seemed to aggregate in an independent cluster.

The present study is one of the first to provide detailed information on the occurrences, co-occurrences, and severity of many different types of potentially addictive behaviors. To assess the problematic occurrence of these behaviors the number of participants above each scale's cut-off threshold were calculated. The highest prevalence estimates of problematic behavior among potentially addictive behaviors were for eating disorders (19.6%). A similar study on randomly selected U.S. university college students measuring eating disorders by the same SCOFF questionnaire found in 2011 that the prevalence of positive screens was lower, 13.5% for women and 3.6% for men (Eisenberg, Nicklett, Roeder, & Kirz, 2011). However, this difference between the rates can be due to changes in the prevalence rates of eating disorders in the past decade. The occurrence of problematic Internet use was 13.3% in the present sample, which is similar to previously reported prevalence rates. According to a recent review of 68 epidemiological studies of Internet addiction (Kuss, Griffiths, Karila, & Billieux, 2014), excessive Internet usage in case of adolescents varied between 0.8% (Italian high school students) and 20.3% (South Korean sample). On a national level, an earlier study has shown on a sample of 1,037 participants that 4.3% of the participants had significant Internet use problems, and 10.1% had some kind of a problematic Internet use (Demetrovics, Szeredi, & Rozsa, 2008). The occurrence rate of problematic online gaming was 4.0% in the present sample, which is similar to previous international (Kuss & Griffiths, 2012) and national prevalence rates as well (Demetrovics et al., 2012; Király et al., 2015; Papay et al., 2013). The occurrence rate of problematic hair pulling was 4.0% in the present sample, which is similar to the prevalence rate reported in a previous Hungarian study comprising over 4,000 participants (Maraz, Hende, Urban, & Demetrovics, 2017). They found that 17% of the sample pulled their hair during the last week, 5% in the past month, 4% in the past year, and 7% over a year ago based on the question of ‘Have you ever pulled your hair?’. Another study emphasize, that although in case of hair pulling, the diagnostic criteria and the clinical prevalence is not clear in the literature, prevalence rate is around 0.6% or using less restrictive diagnosis criteria it might be around 3% (Lejoyeux, McLoughlin, & Ades, 2000), but they also emphasize that this disorder is often unrecognized. Problematic social network use occurred in 3.2% of the present sample, which is in line with a previous national study, where they found that from a representative sample of 5,961 participants 4.5% belonged to the at-risk group on the Bergen Social Media Addiction Scale (Banyai et al., 2017). Occurrence rate of problematic exercising was 2.3% on the present sample, and although there are some inconsistencies in the literature about the prevalence rate of exercise addiction (Egorov & Szabo, 2013), most studies have shown similar prevalence estimates (e.g. Griffiths et al., 2005; Mónok et al., 2012; Terry et al., 2004). The lowest occurrence rate in the present sample was observed in case of problem gambling (1.7%), which is similar to previous prevalence rates. For example, previously a 1.7% prevalence rate was reported as part of the National Survey on Addiction Problems in Hungary (Kun, Balazs, Arnold, Paksi, & Demetrovics, 2012). However, it has to be noted, that the epidemiology of potentially addictive behaviors is hard to conceptualize. In case of most potentially addictive behaviors, research has not yield yet a gold standard classification for normal and problematic usage. Moreover, in some behavioral addiction assessment tools and conceptualizations differ across studies, which make the integration of the results hard to accomplish.

Co-occurrences of the problematic appearance of these behaviors have also been calculated. These analyses (Table 2) suggest that co-occurring problematic behaviors were most frequent among online potentially addictive behaviors (problematic online gaming, problematic Internet use and problematic social networking sites usage), and the highest odds ratios and Phi coefficient values were also observed in these analyses. There is a debate in the literature if these online activities are distinct conceptual and nosological entities or not (e.g. Király et al., 2014). The present co-occurrences underlie the possible interrelatedness of these behaviors, and as such indicate that there might be a substantial overlap between them. However, it is important to note, that although the questionnaires assessing these online activities are specific (e.g. questions of the Problematic Internet Use questionnaire ask about Internet using habits), there is a considerable overlap between some of these behaviors, and it is hard to mentally separate one's habits spending time with these behaviors. Thus, it can not be ruled out that these overlaps might be representing how the participants answer the questions. The strength of the other co-occurrence rates was considerably smaller; however, it is interesting to note, that problematic social networking site usage co-occurred with almost every other problematic behavior (except problematic online gaming) and problematic Internet use, and problematic gambling also showed many significant co-occurrences.

Analyses of the severity of potentially addictive behaviors and regular substance use showed that regular substance users reported higher mean scores on many behavioral addiction scales. Significant associations were found between (i) smoking and problematic Internet use, exercise addiction, gambling, and eating disorders; (ii) alcohol consumption and problematic Internet use, problematic online gaming, problematic social network use, gambling, and eating disorders, and (iii) marijuana consumption and problematic online gaming and gambling. These results are in line with previous reports. For example, an early study showed that adolescent gamblers were more likely to drink alcohol, smoke tobacco, and take illicit drugs compared to non-gamblers (Griffiths & Sutherland, 1998). Similarly, another study of an adolescent sample found that males who used nicotine, alcohol, and cannabis were almost twice more likely to be problematic gamers than non-users (van Rooij et al., 2014). Another recent study (Di Nicola et al., 2015) reported that compared to controls, alcohol use disorder patients had significantly higher scores on scales for gambling disorder, compulsive buying, and sexual addiction. They have also found that individuals experiencing alcohol use disorder with co-occurring behavioral addictions report higher impulsivity and alcohol craving. The key findings of the present study suggest an association between the use of certain substances (especially regular alcohol consumption) and the severity of certain potentially addictive behaviors. Also, some potentially addictive behaviors (problematic Internet use, gambling and eating disorders) appear to associate with substance uses more closely than others (e.g., hair pulling), which might reflect the nature of the present sample (young high school, college and university students), or might suggest that the addictions might be divided into different clusters. More studies are needed to clarify the associations between substance use and behavioral addictions. The group differences in the analyses of substance use and potentially addictive behaviors appeared to be moderately strong and stress the importance of investigating the possible common psychological, genetic, and neural pathways underlying different types of addictions, as suggested in the RDS model (Blum et al., 1996).

In the literature, there have been only a few studies focusing on a wide spectrum of substance use and potentially addictive behaviors, most previous studies focused on the association between substance use and gambling and/or gaming. A very recent study however, investigated the shared associations between self-reported behavioral addictions and substance use disorders and mental health problems (Marmet et al., 2019). Their results showed that behavior addictions and substance use disorders explained between a fifth and a quarter of the variance in severity of mental health problems (major depression, attention-deficit hyperactivity disorder, social anxiety disorder, and borderline personality disorder) and that the individual addictions explained only about half of this explained variance uniquely, the other half was shared between addictions. These results suggest that there might be some common route in the background of the co-occurrence of behavior addictions, substance use disorders and mental health problems. Additionally, a large-scale study (*N* = 9,003) analyzed the co-occurrence of cigarette smoking, alcohol consumption, and gambling and found significant associations between all three behaviors (i.e., greater involvement in one behavior was associated with greater involvement with another such as cigarette smokers being three times more likely to be problem gamblers compared to non-smokers; Griffiths et al., 2010). There is also a small-scale study (*N* = 218), which looked at seven co-occurring behavioral addictions (Andreassen et al., 2013). They found that of the 21 bivariate inter-correlations between the seven behavioral addictions (Facebook addiction, video game addiction, Internet addiction, exercise addiction, mobile phone addiction, compulsive buying, and study addiction) all were positive (and nine significantly). Again, these findings suggest some overlaps between such problematic behaviors. Another study comprising a large representative sample investigated the prevalence of single versus multiple addiction problems and aimed to identify distinct subgroups of people experiencing substance-related and behavioral addiction problems (Konkolÿ Thege, Hodgins, & Wild, 2016). Their findings showed that about half of the adult population struggles with at least one excessive behavior in a given year, and their analyses revealed a higher number of co-occurring addiction clusters than typically found in previous studies. Another study focused on the natural course of addictions (Konkolÿ Thege, Woodin, Hodgins, & Williams, 2015). Their aim was to investigate the chronic vs episodic nature of behavior addictions in a longitudinal design. Their data on prevalence, substance use comorbidity, and five-year trajectories of six excessive behaviors (exercising, sexual behavior, shopping, online chatting, video gaming, and eating) showed that these behaviors are fairly transient for most people. They found that a large majority of people reported having problematic over-involvement for just one of these behaviors and just in a single time period, and they observed a moderately strong decrease in symptom severity across time. In summary, some data are available on the co-occurrences of different types of substance use and behavioral addictions, but most studies either focus only on one or two types of addictions and/or are relatively small-scale. Our data are in line with these previous findings, suggesting that there is a great overlap between the different types of substance use and potentially addictive behaviors.

The present results and these earlier studies underlie the theoretical models which emphasize the phenomenological, symptomological, and neural similarities of different addictions (e.g. the Syndrome Model of Addiction; Shaffer et al., 2004; the Obsessive-Compulsive Spectrum Disorder model by Hollander, 1993; Hollander & Wong, 1995; the Components Model of Addictions by Griffiths 2005; the Reward Deficiency Syndrome by Blum et al., 1996).

A novelty of the PGA study is that it explores the relationships between substance use and potentially addictive behaviors in detail. Earlier studies have mainly focused on the co-occurrences of a few substances and/or potentially addictive behaviors, while the present analyses provided data on the occurrences and co-occurrences of 15 different types of psychoactive substance use, occurrence and co-occurrence of 7 problematic behaviors, and data on the severity of 7 potentially addictive behaviors by regular nicotine, alcohol and marijuana users.

The present study has some limitations. One of these is the convenience nature of the sample and the self-report data, which decreases the generalizability and reliability of the results. Furthermore, the PGA study targeted a very specific age group (i.e., adolescents and emerging adults), therefore interpretations should be treated with caution. Also, when analyzing the occurrence of potentially addictive behaviors, conceptualizing ‘problematic’ and ‘normal’ usage is operationally challenging. In most cases, the normal/problematic cut-off thresholds of the psychometric instruments were based on theoretical and statistical procedures, and the clinical relevance and reliability has yet to be tested. It is also important to note, that we did not test problematic usage in the case of substances (only consumption regularity/frequency). Furthermore, another possible limitation is that the co-occurrences of lifetime substance use can only be interpreted as lifetime co-occurrences. This approach does not allow conclusions to be drawn regarding simultaneous presence of the given substance use: it is possible that the use of substances was consecutive and not parallel. In case of questionnaires of potentially addictive behaviors, most instructions do not specify a specific time frame, the questions are answered in a general manner. Thus, in this case too, conclusions regarding simultaneous presence of problematic behaviors can not be drawn. In summary, we have to note, that the PGA study is a normal population study, investigating the normal spectrum of several substance use and potentially addictive behaviors, and further investigations are needed to clarify these associations in substance use disorders and behavioral addictions. Investigating problematic usage could even provide further information related to the comorbidity of substance and non-substance use related addictive disorders. Furthermore, despite the wide range of potentially addictive behaviors that were assessed, the study did not involve some potentially interesting problematic behaviors (e.g., problematic smartphone use, work addiction, compulsive buying).

Despite these limitations, the present study arguably makes a large contribution to the field and presents epidemiological data from a large-scale national study in Hungary examining a wide range of engagement in potential behavioral addictions and consumption of psychoactive substances. However, due to the nature of the data (convenience sampling), providing prevalence rates of these behaviors was not the focus of the study. Nevertheless, investigating the epidemiological aspects of psychoactive substance use and potential addictive behaviors is important, since occurrence rates could contribute to the conceptualization and classification of normal and problematic appearance of these behaviors. Additionally, analyzing the co-occurrences of these behaviors is a first step in the identification of common psychological, genetic, and neural pathways in association with these behaviors.

## Funding

This work was supported by the Hungarian National Research, Development and Innovation Office (Grant numbers: K111938, KKP126835, NKFIH-1157-8/2019-DT). Eszter Kotyuk was supported by the postdoctoral scholarship of the Hungarian Academy of Sciences. Gyöngyi Kökönyei was supported by the Hungarian Brain Research Program (Grant No. 2017-1.2.1-NKP-2017-00002); ans by the MTA-SE-NAP B Genetic Brain Imaging Migraine Research Group, Hungarian Academy of Sciences, Semmelweis University (Grant No. KTIA_NAP_13-2-2015-0001). Orsolya Király and Bernadette Kun were supported by the János Bolyai Research Scholarship of the Hungarian Academy of Sciences and by the ÚNKP-19-4 New National Excellence Program of the National Research, Development and Innovation Office. Kenneth Blum along with Marjorie Gondre-Lewis (Howard University) are the recipients of G12MD007595/National Institute of Minority Health and Health Disparities.

## Authors' contribution

Study concept and design: ZD, CBdata collection: AM, AE, AV, CB, JF. Analysis and interpretation of data: EK, OK, AV, CB, MDG, AS, RU, KB, ZD. Statistical analysis: EK, RU, obtained funding: EK, GK, OkK, BK, BK. Study supervision: ZD, RDB, RU, KB, CB, MDG, AS, GK. All authors had full access to all data in the study and take responsibility for the integrity of the data and the accuracy of the data analysis.

## Conflict of interest

The authors have no conflict of interest to declare.

## Supplementary Material
