# Supplementary material for: Co-occurrences of substance use and other potentially addictive behaviors: Epidemiological results from the Psychological and Genetic Factors of the Addictive Behaviors (PGA) Study
Source: J Behav Addict. 2020 Jun 26;9(2):272–88. doi: 10.1556/2006.2020.00033 (PMC8939407; doi:10.1556/2006.2020.00033)
Supplement: Supplementary file 1 [file jba-9-272-s001.docx]

**Supplementary Table 1: Differences in lifetime substance use in males and females**

| Lifetime substance use | Sex | | Pearson Chi-square | *p* | OR |
| --- | --- | --- | --- | --- | --- |
|  | Male | Female |  |  |  |
| Cigarettes | 69.7% | 68.7% | 0.346 | 0.557 | 0.954 |
| Alcohol | 95.2% | 94.7% | 0.406 | 0.524 | 0.897 |
| Marijuana | 40.7% | 27.3% | 59.782 | <0.001 | 0.546 |
| Synthetic marijuana | 12.0% | 6.2% | 17.806 | <0.001 | 0.485 |
| Amphetamine | 8.3% | 3.9% | 26.092 | <0.001 | 0.448 |
| Cocaine | 2.9% | 1.0% | 15.165 | <0.001 | 0.334 |
| Heroin | 1.2% | 0.4% | 7.236 | 0.007 | 0.293 |
| LSD | 2.4% | 0.9% | 10.722 | 0.001 | 0.376 |
| Magic mushrooms | 2.8% | 0.8% | 18.087 | <0.001 | 0.282 |
| GHB | 2.4% | 0.8% | 12.966 | <0.001 | 0.329 |
| Mephedrone | 4.9% | 1.7% | 25.088 | <0.001 | 0.335 |
| Steroids | 3.6% | 1.8% | 10.344 | 0.001 | 0.473 |
| Alcohol with drugs | 6.7% | 6.0% | 0.549 | 0.459 | 0.894 |
| Sedatives | 5.1% | 9.7% | 20.834 | <0.001 | 1.975 |
| Other drugs | 3.5% | 1.3% | 16.09 | <0.001 | 0.355 |
| unknown type of drug ^1^ | 2.5% | 1.2% | 5.718 | 0.017 | 0.49 |

^1^ The ‘unknown type of drug’ category represent participants’ answers where they do not know what type of substance they used.

**Supplementary Table 2: Differences in the severity of potentially addictive behaviors in males and females**

| Questionnaires assessing potentially addictive behaviors | Sex | | t-test | p | Cohen's d |
| --- | --- | --- | --- | --- | --- |
|  | male | female |  |  |  |
| Problematic Internet Use Questionnaire (PIUQ) | 10.21 ± 3.5 | 10.22 ± 3.7 | -0.079 | 0.937 | <0.001 |
| Problematic Online Gaming Questionnaire Short-Form (POGQ-SF) | 18.58 ± 7.5 | 13.65 ± 4.0 | 20.936 | <0.001 | 0.856 |
| Bergen Social Media Addiction Scale (BSMAS) | 8.73 ± 3.3 | 10.22 ± 4.0 | -8.452 | <0.001 | 0.397 |
| Exercise Addiction Inventory (EAI) | 12.69 ± 5.1 | 12.24 ± 4.8 | 2.423 | 0.015 | 0.091 |
| Diagnostic Statistical Manual-IV-Adapted for Juveniles (DSM-IV-MR-J) | 0.46 ± 1.2 | 0.11 ± 0.4 | 10.012 | <0.001 | 0.417 |
| The Massachusetts General Hospital Hairpulling Scale (MGH-HPS) | 1.40 ± 3.9 | 1.46 ± 4.1 | -0.293 | 0.769 | 0.016 |
| SCOFF Questionnaire eating disorder questionnaire | 0.49 ± 0.8 | 0.88 ± 1.0 | -11.816 | <0.001 | 0.423 |

**Supplementary material - A list of the questions assessing substance use severity**

Q1. Do you smoke?

0 – No

1 – Yes, occasionally (not every day)

2 – Yes, regularly (every day)

Q2. How many cigarettes do you smoker a day?

1 – 0-5

2 – 6-10

3 – 11-15

4 – 16-20

5 – 21-25

6 – 26-30

7 – 31 or more

Q3. When did you first tried the following substances? (cigarette, regular smoking, alcohol, getting drunk, marijuana, amphetamine, cocaine, GHB, mephedrone)

When I was ………….. years old.

I don’t know

Never

Q4. Last time you drunk, how much alcohol you consumed?

There was a pictogram, demonstrating what is considered as 1 drink (5dl alcohol, 2dl wine, a 0.5dl hard liquor).

0 – I never drunk alcohol

1 – 1 or 2 drinks

2 – 3 or 4 drinks

3 – 5 or 6 drinks

4 – 7 or 9 drinks

5 – 10 or more drinks

Q5. How often did you consume alcohol over the past 30 days?

0 – not once

1 – 1-3 times

2 – 4-9 times

3 – 10-19 times

4 – not every day, but more than 20 times

5 – every day

Q6. How often did it occur in the past 30 days, that you drunk more than 6 drinks on one night?

0 – not once

1 – 1-3 times

2 – 4-9 times

3 – 10-19 times

4 – not every day, but more than 20 times

5 – every day

Q7. How often did you use marijuana in the past 30 days?

0 – not once

1 – 1-3 times

2 – 4-9 times

3 – 10-19 times

4 – not every day, but more than 20 times

5 – every day
